# Esophageal granular cell tumor: Clinical, endoscopic and histological features of 19 cases

**DOI:** 10.3892/ol.2014.2152

**Published:** 2014-05-19

**Authors:** MING ZHANG, ZE-QUN SUN, XIAO-PING ZOU

**Affiliations:** 1 Gastroenterology Department, Drum Tower Hospital Affiliated Medical College of Nanjing University, Nanjing, Jiangsu 210008, P.R. China; 2 Gastroenterology Department, Renmin Hospital, Hubei University of Medicine, Shiyan, Hubei 442008, P.R. China

**Keywords:** granular cell tumor, esophagus, endoscopic resection, endoscopic ultrasound

## Abstract

Esophageal granular cell tumors (GCTs) are rare and often misdiagnosed. To demonstrate their clinicopathological features, the present study reports 19 cases and reviews the literature. There were 11 female and eight male esophageal GCT patients with a median age of 42.0 years. All the tumors were solitary. The majority of patient indications for endoscopy (89.5%) were non-specific and endoscopic therapy was performed in 17 cases with a relapse in one case after a 12-month follow-up. The endoscopic appearance of esophageal GCT was variable and the majority of tumors (80.0%) were located in the middle and lower esophageal segments. The size of the tumors ranged from 0.4 to 2 cm in diameter and the surface was white-gray, pink or yellow. Nine patients underwent an endoscopic ultrasound exam, eight of which demonstrated hypoechoic echostructures with a smooth margin and intracavity growth features. One case was derived from the muscularis propria layer with an irregular margin and intra- and extra-cavity growth features. The histological features could mimic other tumors and immunohistochemical stains are usually positive for S-100, periodic acid-Schiff, neuron-specific enolase and nestin. Three cases indicated pleomorphism and Ki-67 was locally positive. Esophageal GCTs are rare and endoscopic ultrasound features are variable. Immunohistochemical staining may aid in the diagnosis.

## Introduction

Granular cell tumor (GCT), which was first reported by Abriksoosoff in 1926 (1), is an uncommon and usually benign tumor (2). It occurs in almost all organs, but mostly in the skin or soft tissues. GCT may occur in one tissue, and in certain cases in multi-organs simultaneously or asynchronously (2). Currently, it is widely accepted that GCT derives from neurogenic Schwann cells (3). GCT occurring in the gastrointestinal tract accounts for only 8% of all GCTs (2). Esophageal GCT, which was also first reported by Abriksoosoff in 1931 (4), is the most common type of GCT in the gastrointestinal tract. It is estimated that esophageal GCT accounts for approximately one-third of all GCTs in the gastrointestinal tract and ~1% of all esophageal benign tumors (5–7). Esophageal GCTs exhibit almost the same clinicopathological characteristics as GCTs of other organs. Although the diagnosis of GCT is relatively straightforward, deciding on an appropriate treatment strategy is often complex. Previously surgical resectioning, including wide excision, was the recommended treatment strategy (8). However, in recent years this technique has been gradually abandoned (5). Currently, the majority of GCT patients undergo endoscopic resectioning, after which, the majority of tumors are removed and relapse is rare either *in situ* or in other locations even after several years. However, relapse seldom affects the patients’ lifespan (5).

Since the first report of GCT, ~300 esophageal GCTs have been reported in literature (3). The aim of the present study is to describe their clinical, endoscopic and histological features.

## Materials and methods

All the data were collected in our endoscopic centers (Digestive Endoscopy Center, Drum Tower Hospital Affiliated Medical College of Nanjing University, Nanjing, China; and Digestive Endoscopy Center, Renmin Hospital, Hubei University of Medicine, Shiyan, China) between January 2005 and June 2013. Following the identification of esophageal GCT, patient demographic data, which includes age, gender, indications for endoscopy, therapeutic methods, the records of colonoscopy and endoscopic ultrasound (EUS), were carefully collected. Endoscopic and EUS images were reviewed again by two of our authors. The follow-ups were conducted by calling the patient and asking for their health condition, including if there were new tumors, relapse or metastasis of the primary tumor. All the patient slides were checked again for histological and immunohistochemical stains. The protocol of the present study was prepared according to the Declaration of Helsinki and approved by the ethics committee of the Medical School of Nanjing University and Hubei University of Medicine (China).

## Results

During the survey period, 19 cases (11 female and eight male) of esophageal GCTs were identified. The median age at the time of diagnosis was 42.0 years old (range, 24–71 years old). For all the cases, the tumor was solitary. The majority of patient indications for endoscopy were non-specific. Two patients with a tumor diameter of ≥1.5 cm complained of dysphagia. Following the removal of the tumor, the symptom of dysphagia was relieved. Endoscopic therapy was effectively performed in 17 cases, and no complications occurred during the procedure or in the following period. Colonoscopy was undertaken in nine patients and no colonic GCT was identified. The median follow-up period was 45 months (range, 7–95 months). During the follow-up period, one patient was lost to follow-up and one confirmed another esophageal GCT after 12 months. An endoscopic forcep biopsy was obtained from five of the 19 patients and three confirmed the diagnosis of GCTs. The general clinical information is presented in Table I.

**Figure 1:**
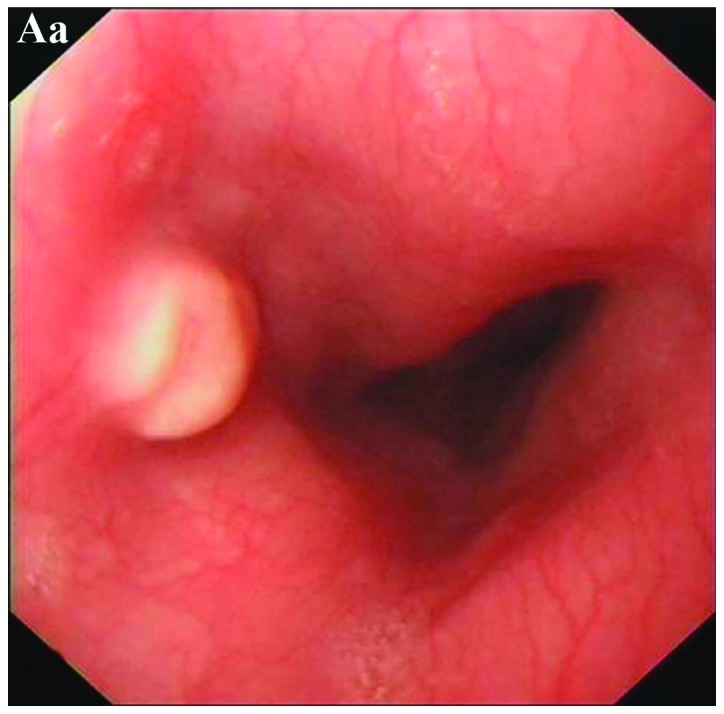

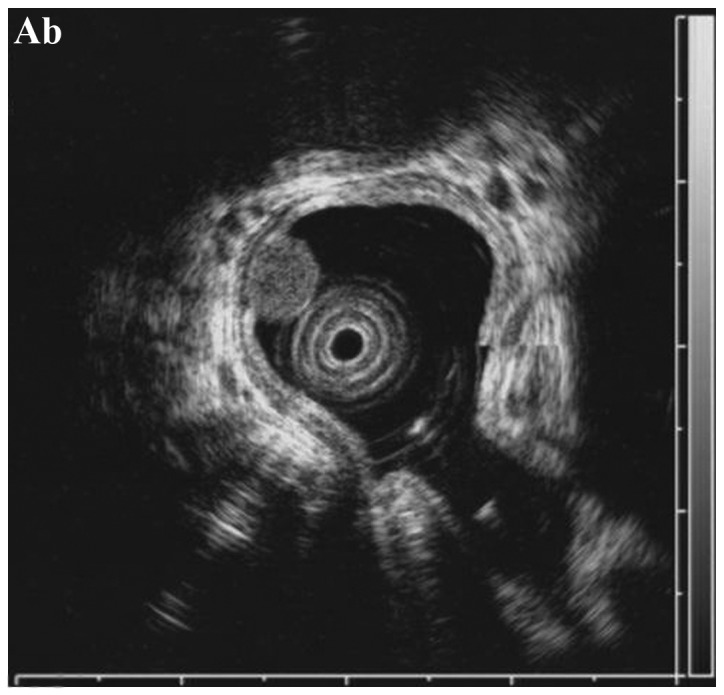

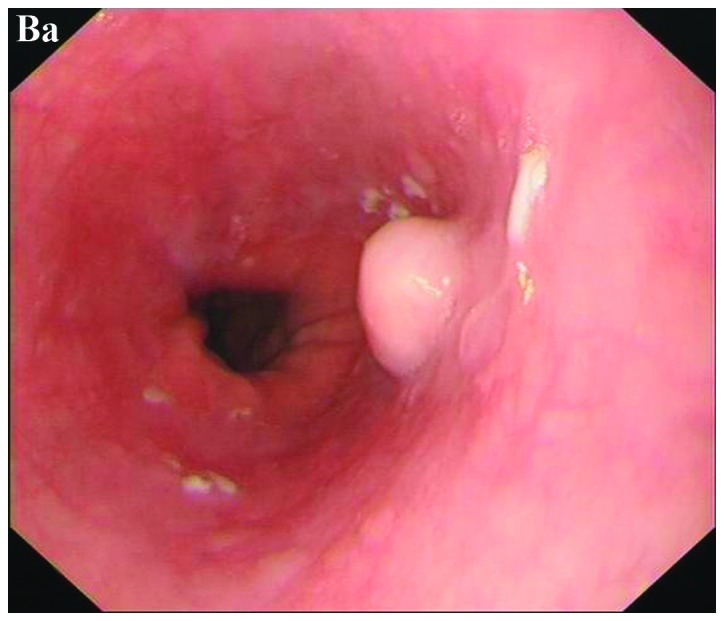

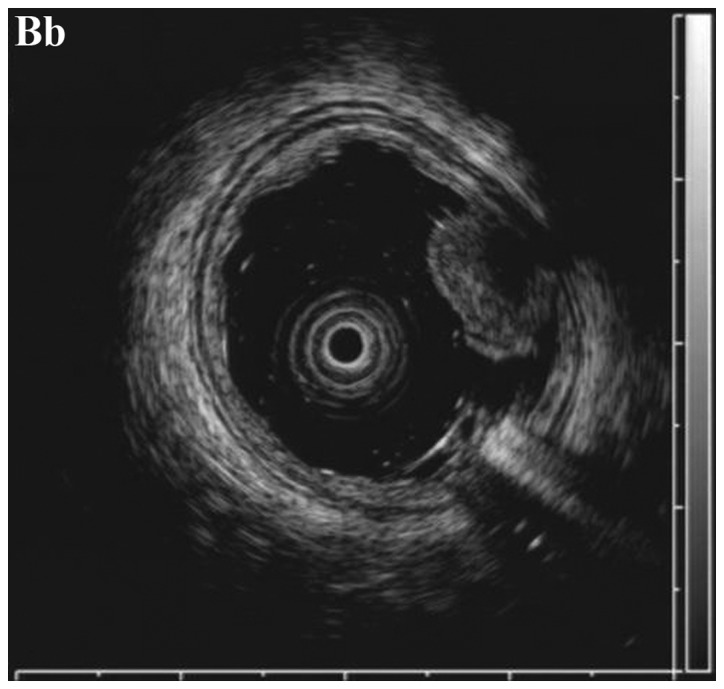

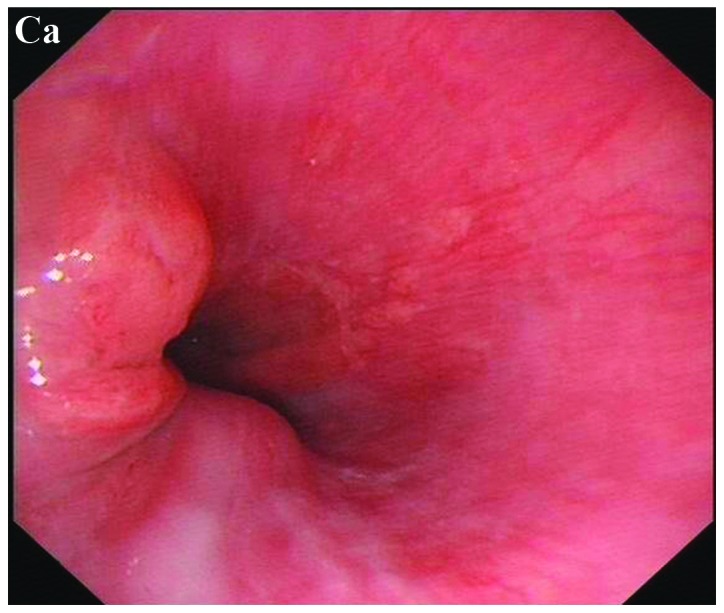

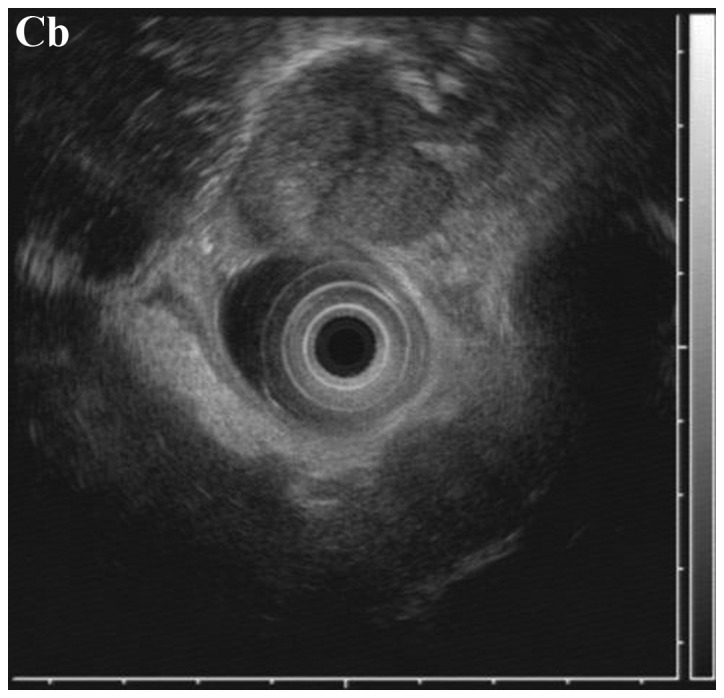
Endoscopic and EUS images of esophageal granular cell tumors. (Aa) A white-to-gray tumor with a smooth surface and (Ab) the EUS image of the same patient showing hypoechoic, homogeneous, smooth-edged lesions derived from mucosal layers. (Ba) A white tumor with a smooth surface and (Bb) the EUS image of the same patient showing hypoechoic, heterogeneous, smooth-edged lesions derived from mucosal layers. (Ca) A pink tumor with a smooth surface and (Cb) the EUS image of the same patient showing hypoechoic, irregular margin and intra- and extra-cavity growth. EUS, endoscopic ultrasound.

The endoscopic appearance of esophageal GCT was variable. All 20 tumor surfaces were smooth and white to gray (n=9), pink (n=7) or yellow (n=4) in color (Fig. 1). The primary endoscopic diagnoses were GCT (n=7), polyp (n=6), leiomyoma (n=4), lipoma (n=2) and interstitialoma (n=1). Tumors were located in the upper, middle and lower segment of the esophagus in four, eight and eight cases respectively. The tumor size ranged from 0.4 to 2.0 cm, with a median size of 0.7 cm. One patient had the occurrence of two tumors, which were located in the middle segment of the esophagus with similar endoscopic features.

Nine patients underwent EUS by UM-20-29R (12 MHz) or UM-2R (20 MHz), eight of which demonstrated a smooth margin with intracavity growth features. Tumors were derived from the mucosa, muscularis mucosa, submucosa layer and muscularis propria layer in two, four, two and one cases, respectively (Fig. 1). The case that derived from the muscularis propria layer indicated an irregular margin and intra- and extra-cavity growth features. The majority of the tumors demonstrated hypoechoic (n=8) echostructures. A total of six and three cases indicated homogenous and heterogeneous echoic features, respectively. No necrosis or fibrosis was found inside the tumors with heterogeneous echoic by histological examination.

**Figure 2:**
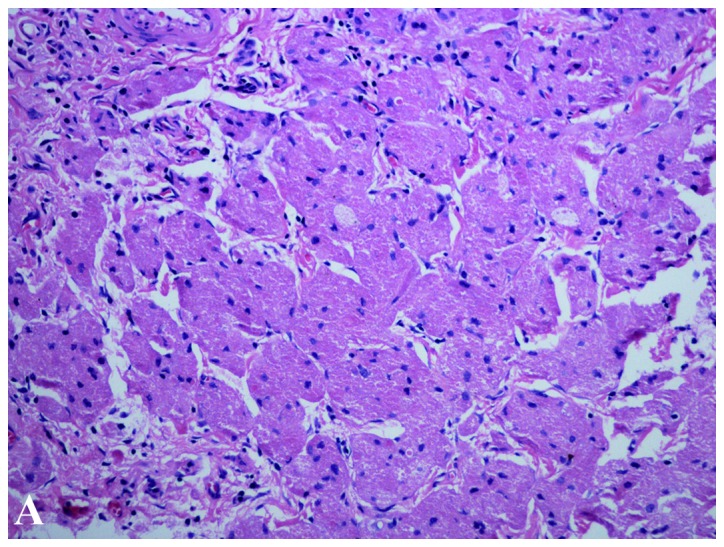

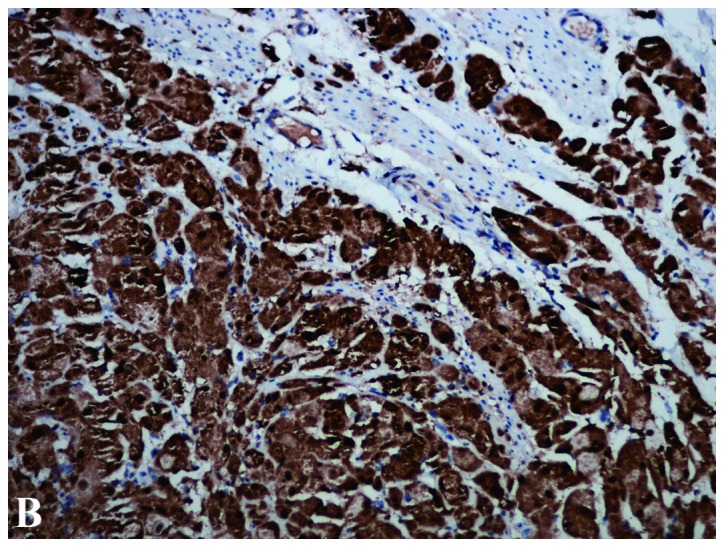

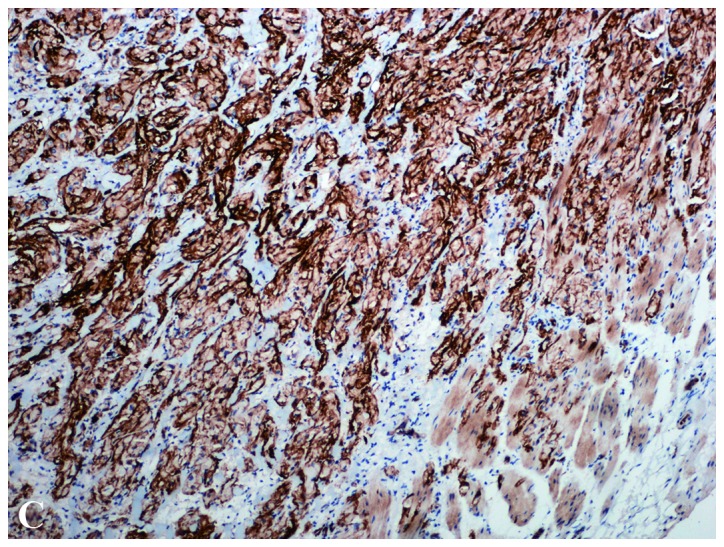
Histological and immunohistochemical images of esophageal granular cell tumors. (A) The tumor indicates a nest appearance with an obscure boundary, separated by fibers and infiltrating surrounding layers. The cytoplasm contains eosinophilic granules with round nuclei. No necrosis or nuclear fission was determined (hematoxylin and eosin stain; magnification, ×200). (B) The cytoplasm and nuclei of the tumor cells show positive immunostaining for S-100 protein (immunoperoxidase; magnification, ×200). (C) The tumor cells show nestin-positive staining (immunoperoxidase; magnification, ×100).

Histologically, the tumors demonstrated a nest appearance, which was separated by fibers with non-capsulated margins and obscure boundaries. The tumor cells could be spherical, polygon or fusifom in shape with infiltration of the surrounding layers. The cytoplasm contained abundant eosinophilic granules and a tiny round nucleus located centrally. No necrosis or nuclear fission was determined in the tumor cells (Fig. 2). Pleomorphism was identified in three cases and 16 of the 19 cases underwent immunohistochemical staining. All 16 cases indicated positive staining for S-100, periodic acid-Schiff-diastase (PAS-D) and nestin, and negative staining for cluster of differentiation 117 (CD117), CD34, desmin, cytokeratin (CK) and smooth muscle antibody (SMA) (Fig. 2). A total of 12 of the 16 cases were CD68-positive. Of the three cases that indicated pleomorphism, the percentage of Ki-67-positive cells was >2%. All the other cases were Ki-67-negative. The two tumors that occurred in the same patient showed the same histological and immunohistochemical stain features.

## Discussion

GCTs, once referred to as Abrikossoff’s tumors or granular cell myoblastomas (1,2), are relatively uncommon and esophageal GCT is much rarer (4). With the widespread use of gastroscopy, more frequent detection of this tumor type has become possible and endoscopic tumor excision is becoming more frequent (5,8).

Esophageal GCT could occur at any age, but it is more common in 40- to 60-year-old patients (9,10) and in females compared with males (5,10–12). The present study reconfirmed these results. Thirty years ago, studies of esophageal GCTs were mainly derived from the symptom of dsyphagia and autopsy (11). However, the majority of esophageal GCTs have been diagnosed incidentally in more recent years, due to the widespread usage of endoscopy (2,12). If the tumor size is ≥1 cm, the patients may complain of dysphagia (5,11). Similar to most studies in recent years, the majority of cases in the present survey were not diagnosed due to dysphagia. In the 2 patients who complained of dysphagia, the tumor size was ≥1.5 cm.

GCTs may occur multiple times in one tissue type and in multiple organs (2). It has been previously reported that ~5–30% of esophageal GCTs were multiple (2,5,12). In the present study, there were no multiple esophageal GCTs or GCTs in other organs. Only one new esophageal GCT was identified during the 12 months follow-up in one patient. Zhong *et al* (5) reported that ≥80% (19/23) of patients could be diagnosed by endoscopic forcep biopsy. At the Digestive Endoscopy Centers of Drum Tower Hospital Affiliated Medical College of Nanjing University and Renmin hospital Hubei University of Medicine, biopsies were only performed when the tumor size was ≤0.6 cm. If the tumor size was >0.6 cm, EUS and endoscopic resection were recommended. Ultimately, in the present study only five patients underwent biopsy and three were diagnosed with GCT.

Typically, the endoscopic feature of esophageal GCT is an elevated lesion with a white-to-gray appearance (3,5). However, certain tumors may show a pink or yellow appearance (3,5). The surface is usually smooth and, in certain cases, coincides with ulceration or necrosis (3). The tumors are usually located in the middle and lower segments of the esophagus. The results of the present study showed similar endoscopic features to such previous studies.

The first and largest study reporting the EUS features of esophageal GCT was published in 1997 (12). It concluded that esophageal GCTs show a hypoechoic and homogeneous echostructure that usually derives from mucosa and muscularis mucosa layers, and has smooth edges (12). Various results were reported in other studies (3,4,12,13). Hyperechoic, besides one case in the present study, has also been reported in certain cases (5). This type of feature usually causes the misdiagnosis of lipoma, particularly in patients with tumors that have a yellow surface. Even though homogeneous echostructure and smooth margins are often reported (12,13), heterogeneous echostructure and irregular margins have also been encountered (5). The causes of these differences remain unknown. Due to all these atypical features, a negative attitude is held for the advantages of EUS in surveillance of esophageal GCTs (12). However, considering extra-cavity growth (one case in the present study) and safety of resection, simple endoscopic surveillance may not be sufficient. EUS surveillance should be undertaken during follow-up and prior to endoscopic resection. Allowing for the fact that the majority of GCTs grow extremely slowly, if no new symptoms appear then a two-year interval is adequate.

The first report of endoscopic therapy for esophageal GCT was in 1979 (14). Since then, endoscopic therapy, which includes endoscopic diathermic electrosurgical snare, endoscopic polypectomy, endoscopic mucosal resection and endoscopic submucosal dissection, for esophageal GCTs has become increasingly popular (3,5). In the present study, 17 of the 19 cases underwent endoscopic therapy safely. Even though it is a benign disease, certain tumors may undergo a malignant change and some tumors may reoccur following resection (15,16). Follow-up was indicated for all the patients, independent of tumor resection (15,16). In the largest series as yet, specific cases left untreated showed either a stable tumor size or regression of the tumor (9).

Histological features of esophageal GCTs could mimic esophageal squamous cancers (17), particularly spindle-cell squamous cancers. In certain cases these two diseases could co-exist in the same patient (18,19). The most differentiating points between them are the nuclear-cytoplasmic ratios and nuclear fission. For esophageal GCT the nuclear-cytoplasmic ratios are usually low and nuclear fission is rare. However, for malignant esophageal GCT, it is extremely difficult to differentiate it from esophageal squamous cancers. Besides, GCT can also mimic gastrointestinal stromal tumor (GIST) (20) or leiomyoma. In the present study, two of the five patients who underwent biopsy were misdiagnosed prior to resection; one was misdiagnosed as GIST and the other as leiomyoma.

If histological features cannot aid the diagnosis, immunohistochemical staining may be helpful. Since the positive staining of S-100 was first reported in 1986 (21), other positive markers have also been reported, including PAS (22), neuron-specific enolase (19) and nestin (7). Negative markers, which include CD117, CD34, desmin, CK, SMA, glial fibrillary acidic protein, inhibin-α, myoglobin, fibronectin and carcinoembryonic antigen, have also been reported (7,23–25). All these markers will aid the differential diagnosis of GIST, leiomyoma and squamous cancers. Ki-67, which is a nuclear proliferation-associated antigen and is considered as a reliable marker of cell proliferation, was reported negative in the majority of cases, however, in certain cases it was locally positive (25,26). In the present study, Ki-67 was identified as locally positive in three cases and, in these cases, pleomorphism was observed. Thus, it is indicated that Ki-67 staining should be undertaken in all GCTs to identify the differential degree of the tumor.

In conclusion, esophageal GCTs occurred mostly in females between 40 and 60 years old, and almost all were solitary in the present study. The endoscopic and EUS features varied widely and EUS may be necessary in the surveillance. Immunohistochemical staining of tumor markers will aid the diagnosis and differential diagnosis.

## Figures and Tables

**Table I:** Clinical characteristics of the 19 esophageal GCT cases.

Clinical characteristics	n	%
Gender		
Female	11	57.9
Male	8	42.1
Indications for endoscopy		
Epigastric discomfort	5	26.3
Abdominal distention	3	15.8
Heart-burn	6	31.6
Epigastric pain	3	15.8
Dysphagia	2	10.5
Therapeutic methods		
Endoscopic polypectomy	12	63.2
Endoscopic mucosal resection	2	10.5
Removed by biopsy forcep	3	15.8
Surgery	1	5.3
Untreated	1	5.3

GCT, granular cell tumor.

## Abbreviations

GCT: granular cell tumor
EUS: endoscopic ultrasound
SMA: smooth muscle antibody
